# Current Approaches to Airway and Ventilation Strategies in Laryngotracheal Surgery: A Narrative Review

**DOI:** 10.3390/medicina61122208

**Published:** 2025-12-15

**Authors:** Roberto Giurazza, Antonio Corcione, Rosanna Carmela De Rosa, Giuseppe Tortoriello, Francesco Coppolino, Vincenzo Pota, Francesca Piccialli, Pasquale Sansone, Maria Beatrice Passavanti, Maria Caterina Pace

**Affiliations:** 1 Department of Woman, Child, General and Specialized Surgery, University of Campania “Luigi Vanvitelli”, Via Luigi de Crecchio 2, 80138 Naples, Italy; francesco.coppolino@unicampania.it (F.C.); vincenzo.pota@unicampania.it (V.P.); francesca.piccialli@studenti.unicampania.it (F.P.); pasquale.sansone@unicampania.it (P.S.); mariabeatrice.passavanti@unicampania.it (M.B.P.); mariacaterina.pace@unicampania.it (M.C.P.); 2 Department of Critical Care, AORN Ospedali dei Colli, Via Leonardo Bianchi, 80131 Naples, Italy; antonio.corcione@ospedalideicolli.it (A.C.); rosanna.derosa@ospedalideicolli.it (R.C.D.R.); 3 Division of Ear, Nose and Throat Surgery, AORN Ospedali dei Colli, Via Leonardo Bianchi, 80131 Naples, Italy; giuseppe.tortoriello@ospedalideicolli.it

**Keywords:** airway management, laryngotracheal surgery, artificial respiration, anesthesia, laryngostenosis

## Abstract

*Background and Objectives*: Airway management and ventilation during laryngotracheal surgery represent some of the most challenging tasks in anesthesiology. The shared airway between the surgeon and anesthesiologist requires continuous coordination to ensure optimal oxygenation while maintaining an unobstructed surgical field. *Materials and Methods*: This narrative review is based on a comprehensive literature search of PubMed, Embase, Scopus, and Google Scholar, covering all publications from inception to 30 June 2025. The literature search was performed using a defined Boolean strategy and explicit inclusion/exclusion criteria, focusing on adult human subjects. The search included combinations of the terms “laryngotracheal surgery,” “airway management,” “ventilation strategies,” “jet ventilation,” “Tritube,” and “Flow Controlled Ventilation.” Only English-language studies focused on human subjects were included. *Results*: Traditional ventilation strategies, such as apneic oxygenation and jet ventilation, remain widely used but present limitations in terms of gas exchange efficiency, risk of barotrauma, and surgical interference. In recent years, new devices and ventilation modes—particularly the Tritube^®^ combined with Flow-Controlled Ventilation—have emerged as promising alternatives. These approaches allow continuous ventilation with minimal airway diameter, improving surgical access and patient safety. FCV’s potential to optimize gas exchange and reduce mechanical power is physiologically compelling, but its supporting evidence remains limited and heterogeneous, primarily consisting of small, single-center studies and case series. *Conclusions*: Optimal airway and ventilation management in laryngotracheal surgery requires individualized planning, technical expertise, and close interdisciplinary communication. This approach must integrate objective neuromuscular monitoring to ensure patient safety and include a comprehensive strategy for safe postoperative airway management and extubation. While emerging technologies have significantly expanded available options, their successful application depends on training, experience, and appropriate case selection. Further high-quality clinical studies are needed to standardize protocols and validate long-term outcomes of these innovative ventilation strategies.

## 1. Introduction

Securing an airway and ensuring proper ventilation of the patient’s lungs during laryngeal and tracheal surgery can be a significant challenge for the anesthesiologist for several reasons [1].

This difficulty stems from the complex interaction between the need to manage a shared airway and the frequent presence of a difficult airway, often complicated by an infiltrating tumor, scarring, or edema. Successful endoscopic procedures, such as transoral laser microsurgery (TOLMS), critically depend on optimal surgical exposure, especially in the case of lesions in the posterior commissure [2]. The consequent need for small-bore endotracheal tubes (ETT)—necessary both to intubate a narrowed airway and to grant better surgical exposure—in most cases hinders safe and efficient lung ventilation.

Moreover, since the main indication for endoscopic laryngotracheal surgery is cancer and since heavy smoking is a primary risk factor for head and neck cancers, these patients often suffer from multiple comorbidities, especially chronic obstructive pulmonary disease (COPD) and emphysema with bronchial hyperreactivity, which further impede adequate lung ventilation. Indeed, such diseases determine an increase in airway resistance and lung elastance, which can sometimes require prioritizing patient’s ventilation over optimal surgical exposure.

Beyond airway access and ventilation challenges, laryngotracheal surgery itself is technically demanding. Indeed, surgical procedures involving delicate structures in the upper airway can be complicated by swelling and bleeding [3]. The use of carbon dioxide (CO_2_) lasers adds further risks, including fire and explosion, requiring careful selection of materials, techniques, and oxygen delivery strategies to minimize this risk [4].

Over the years, different strategies for airway management have been proposed for laryngotracheal surgery, including microlaryngeal tubes, high-frequency jet ventilation (HFJV), supraglottic superimposed high-frequency jet ventilation (SSHFJV), and laser-safe small-bore oral ETTs.

Although these approaches aim to maximize the surgical field, their integration with conventional ventilation techniques often proves insufficient in patients with COPD, who are particularly prone to dynamic hyperinflation, leading to progressive increase in airway resistance and airway pressures, barotrauma, hypoventilation, hemodynamic instability, and even cardiovascular collapse if left untreated.

These limitations have prompted the exploration of alternative solutions, including apneic oxygenation and spontaneous breathing techniques, as well as the ultrathin cuffed ETT (Tritube^®^ by Ventinova Medical, Eindhoven, The Netherlands), coupled with a dedicated ventilator supporting the Flow-Controlled Ventilation (FCV) modality.

This narrative review aims to address the key challenges in airway and ventilation management during laryngotracheal surgery. Specifically, it seeks to answer the following questions: (1) What are the most frequent airway-related difficulties encountered in this setting? (2) What are the advantages and limitations of traditional and modern ventilation techniques? (3) How can recent innovations, such as the Tritube^®^ and FCV, improve safety and outcomes for both patients and surgical teams? By exploring these issues, this review provides a comprehensive overview of current practices and highlights emerging strategies to optimize perioperative airway management.

## 2. Materials and Methods

This narrative review is based on a comprehensive literature search of electronic databases, including PubMed, Embase, Scopus, and Google Scholar, covering all publications from inception to 30 June 2025. To ensure reproducibility, the following Boolean search string was employed: (“laryngotracheal surgery” OR “laryngeal surgery” OR “airway surgery”) AND (“airway management” OR “ventilation strategies” OR “jet ventilation”, OR “high-frequency jet ventilation” OR “apneic oxygenation”) AND (“Tritube” OR “Flow Controlled Ventilation” OR “FCV” OR “microlaryngeal tube”). The literature search was focused on articles highly relevant to perioperative airway and ventilation management in laryngotracheal surgery. We included (1) studies involving adult human subjects, (2) articles specifically addressing ventilation techniques and devices, (3) articles published in English, (4) guidelines, systematic reviews and meta-analyses, and original articles and case series with associated review of literature. We excluded (1) studies involving the pediatric population, (2) conference abstracts, (3) papers lacking direct relevance to anesthesiology.

The selection of articles was performed based on relevance to the review’s objective, and additional references were identified by manually screening the bibliographies of key papers. Due to the necessary heterogeneity of the literature consulted (including technical reports, case series, and randomized trials), the authors determined that a formal, quantitative risk-of-bias assessment (such as RoB 2.0 or GRADE) would be methodologically inappropriate for the scope of this narrative review. To ensure the quality and rigor of this narrative review, the methodological process was formally assessed using the Scale for the Assessment of Narrative Review Articles (SANRA), achieving a final score of 12 out of a possible 14 points [5].

## 3. Preoperative Assessment and Airway Management Algorithms

The American Society of Anesthesiologists (ASA) defines a difficult airway as an anticipated or unanticipated difficulty or failure by an experienced physician in anesthesia care in one or more of the following: facemask ventilation, laryngoscopy, ventilation using a supraglottic device, tracheal intubation, extubation, or creation of an invasive airway [6]. This broad definition emphasizes that the difficulty is not limited only to difficult tracheal intubation.

In ear, nose, and throat (ENT) surgery, especially in upper airway surgery, a difficult airway is encountered most of the time.

A thorough pre-anesthetic airway risk assessment is mandatory and should be performed by the anesthesia provider in charge of the patient, including evaluation of the airway patency and diameter through preoperative imaging (computed tomography or magnetic resonance imaging), as well as indirect or fiberoptic laryngoscopy, sharing clinical information with the surgical team. This allows the anesthesiologist to prepare all the necessary equipment and choose the preferred strategy for securing the airway [7].

Should there be any risk factors, such as an anticipated difficulty in facemask or supraglottic airway ventilation, an increased risk of aspiration or rapid desaturation, or a suspected difficult emergency invasive airway, the anesthesiologist should consider an awake tracheal intubation technique, including awake fiberoptic intubation (AFOI), video laryngoscopy, and retrograde intubation [6]. In case there are no risk factors, the anesthesiologist can proceed with intubation attempts after induction of anesthesia. In either scenario, preoxygenation should be ensured and optimized with low-flow or high-flow nasal cannulas (HFNC) during attempts to secure an ETT [8,9,10].

In the event of difficult tracheal intubation after induction of anesthesia, the anesthesiologist should always call for help, limit the number of attempts to a maximum of three—plus one performed by a senior provider—and must always ensure adequate ventilation by any airway technique. If ventilation and oxygenation are sufficient, it is a non-emergency situation, where the patient can be awakened or assisted with a supraglottic airway or other intubation device. A “cannot intubate cannot oxygenate” (CICO) situation occurs after attempts to manage the airway through facemask, supraglottic airway, and intubation have failed, and neither ventilation nor oxygenation is possible. This is an emergency condition where profound hypoxia and oxygen desaturation can lead to hypoxic brain injury, cardiac arrest, or death if untreated. In such a crisis, an emergency surgical airway must be rapidly established through a cricothyroidotomy or tracheostomy as a fundamental lifesaving maneuver [6].

## 4. Airway Management Devices

Although optimal surgical exposure is critical for the success of endoscopic laryngotracheal surgery, such as TOLMS [2,11], the surgical need for a wide, clear, and spacious field may contrast with the need to protect the airway and provide adequate oxygenation and CO_2_ removal to the patient [12].

In addition, technical difficulty may be further increased by the presence of glottic or tracheal stenosis. In such cases, a conventional 7.5 mm internal diameter (ID) ETT could be impossible to place, and, if placed, it would impede surgical access to the lesion by occupying most of the space available in the airway [13].

Over the years, different devices and techniques have been proposed to address this issue and optimize surgical exposure, including HFJV, intermittent apneic oxygenation, spontaneous breathing techniques, modern cuirass ventilation, microlaryngeal tubes (MLTs), laser-resistant small-bore tracheal tubes, and, recently, ultrathin ETTs. Each of these has some advantages but also comes with its drawbacks.

Jet ventilation (JV) is based on the delivery of an intermittent high-pressure gas flow through a jet nozzle in the upper airway using a small catheter. In JV, there is no seal between the delivery system and the airway. Hence, the expiration of gas around the jet catheter is passive and requires a patent upper airway with adequate time between jet insufflations to avoid dynamic hyperinflation and barotrauma. In JV, oxygenation and CO_2_ removal mostly depend on gas diffusion in the bronchial tree. A variation of this technique is HFJV, in which very small tidal volumes are delivered at high pressure and high frequency (100–150 jets per minute), to improve diffusion efficacy. According to the position of the jet nozzle, there are three variants of JV: supraglottic (the jet nozzle is above the vocal cords), infraglottic (a small-caliber jet catheter is passed through the vocal cords), and transtracheal (flow is delivered through a cricothyroid cannula) [14]. A particular type of HFJV is SSHFJV, where the jet nozzle is integrated into the surgical laryngoscope [15]. HFJV is useful in procedures where an ETT would impede or limit surgical exposure of the airway, when anesthetic equipment could be damaged by surgical maneuvers, in the case of rigid bronchoscopy, or ventilation through a cricothyroidotomy. Nevertheless, despite its historical utility in providing a clear and optimal view of the surgical field, HFJV has the disadvantages of not protecting the airway, with risk of aspiration and inhalation of fumes and surgical particles, which can also be dispersed in the operating room. Importantly, HFJV also has some relevant physiological limitations, primarily related to a lack of controlled expiratory flow and potential for gas trapping. Indeed, some difficulties can be encountered in oxygenation and CO_2_ removal in patients with morbid obesity, restrictive and obstructive pulmonary disease, and impaired lung diffusion capacities [12], resulting in hypoxemia and severe respiratory acidosis with eventual abortion of the surgical procedure. There is also a high risk of barotrauma and lung hyperinflation, due to the high pressure of the jet stream and the need for prolonged expiratory times in COPD patients [11]. Other complications include pneumothorax and pneumomediastinum, laser burns to the trachea, mucosal dehydration, and stomach distension with risk of regurgitation [16]. The challenges associated with reliable monitoring and gas exchange stability in HFJV have driven the search for alternative technologies that offer more precise flow control.

The intermittent apnea technique consists of glottis exposure through direct laryngoscopy, with repeated phases of intubation and extubation. Laser endoscopic surgery is performed after removal of the ETT during the apneic intervals, whose duration depends on the patient’s tolerance, oxygen saturation, and transcutaneous CO_2_ monitoring. To prolong the operating phase, it is possible to perform apneic oxygenation through HFNC [17]. Of course, the main complications include hypoventilation, hypercapnia, oxygen desaturation, atelectasis, aspiration, and laryngospasm [18].

An alternative is the spontaneous ventilation technique, where the patient is anesthetized but not paralyzed, therefore being able to breathe autonomously. An oxygen source is integrated in the side port of the operating laryngoscope. It is also possible to use HFNC in order to optimize gas exchange; this technique is known as THRIVE (Transnasal Humidified Rapid Insufflation Ventilatory Exchange) and has been shown to facilitate oxygenation and CO_2_ removal through gaseous mixing and flushing of dead space [8]. In this approach there is unobstructed access to the larynx, although there is no protection against aspiration and operating room pollution with a high risk of laryngospasm [19]. The associated complications are the same as those of the previous technique.

MLTs are standard cuffed polyvinyl chloride (PVC) ETTs, with an ID ranging from 4.0 to 6.0 mm and a longer length, facilitating insertion in narrowed airways and offering surgeons a clearer view and more space in the surgical field. They are not suitable for laser surgery.

Laser-resistant tubes are cuffed, non-flammable, flexible, and stainless steel corrugated spiral ETTs, suitable for CO_2_ and potassium titanyl phosphate (KTP) laser beams, with an ID ranging from 4.5 to 6.0 mm. They have an airtight steel spiral that prevents air leaks occurring along the tube’s length and two extra-large tracheal cuffs (proximal and distal). These ETTs provide significantly superior protection against ignition compared with standard PVC ETTs. The ASA recommends the use of laser-resistant ETTs for upper airway surgery involving the use of laser beams. The tube cuff should be filled with sterile saline mixed with methylene blue, since this could be an indicator in case of cuff rupture [4]. Of course, during laser surgery, the delivered fraction of inspired oxygen (FiO_2_) should be kept as low as possible, conventionally below 30% to reduce the risk of accidental ignition [14].

Both laser-resistant ETTs and MLTs have a small caliber, allowing protection of the airway, as well as ventilation of the lungs, while offering only minimal impediment to the surgical endoscopic procedure. One of the limitations is that they obstruct the vision of the posterior commissure, often necessitating anterior dislocation of the ETT by the surgeon to allow complete resection (R0) of the lesion. Importantly, given their tiny ID, the main issue for the anesthesiologist in charge is ventilation, with significant risk of air trapping, dynamic hyperinflation, increased airway pressure, hypoventilation, hypercapnia, hypoxia, barotrauma, and hemodynamic decompensation. These issues will be further analyzed in the next section of the article.

So far, the presented airway devices have shown the compromise that surgeons and anesthesiologists, competing for the airway, have constantly been facing: accepting an inadequate view of the glottis to properly ventilate the patient, or conversely, accepting inadequate ventilation of the patient to optimize the exposure of the surgical field.

Cuirass ventilation consists of the application of an airtight shell around the chest and abdomen of the patient, which allows non-invasive ventilation of the lungs by applying phases of negative and positive pressure inside the cuirass. This has the advantage of providing unlimited access to the non-intubated airway for surgery, as well as adequate gas exchange, without the risk of hypercapnia associated with apneic techniques. The main disadvantages of this technique in laryngotracheal surgery include non-protection of the airway, glottic movements associated with air passage, and operating room contamination [20,21].

**Figure 1:**
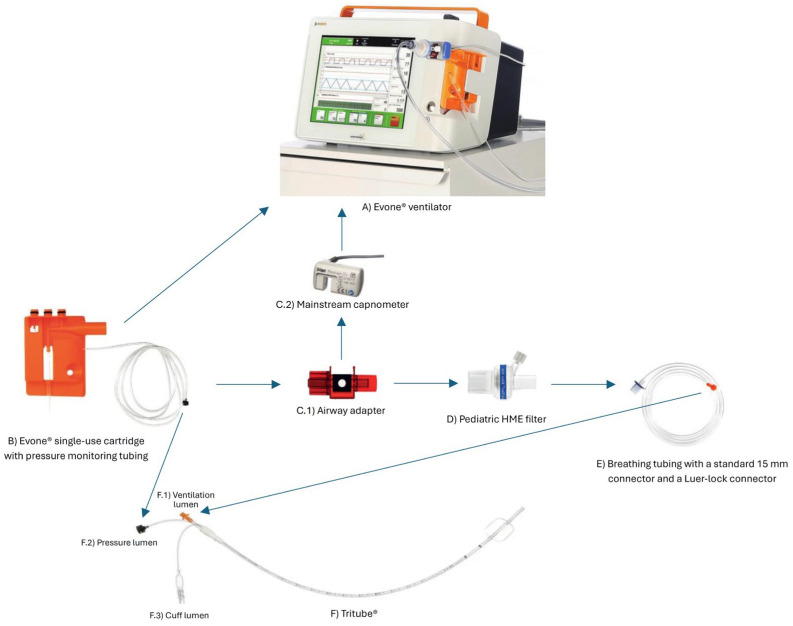
This diagram shows the Tritube® setup with its dedicated Evone® ventilator and specific connectors and consumables.

A recent development in airway management for laryngotracheal surgery is the availability of ultrathin cuffed endotracheal tubes, among which the Tritube^®^ is one example. With an ED of 4.4 mm and an ID of 2.4 mm, this device aims to facilitate intubation in patients with airway stenosis (also with AFOI), while maintaining a relatively unobstructed surgical field. Moreover, it may be well tolerated by patients during awake extubation strategies [22]. It may be indicated in the case of severe glottic or subglottic stenosis or in the case of difficult ventilation with other conventional devices. The Tritube^®^ incorporates three lumens: a primary lumen for ventilation (with a Murphy eye and Luer Lock connector), one dedicated to continuous airway pressure measurement, and one for cuff inflation. Notably, the Tritube^®^ is not laser-certified; therefore, protective measures such as wet gauze shielding are required during laser surgery [12]. Because of its Luer Lock connection and its geometric characteristics, the device is compatible only with specific ventilation systems (e.g., Ventrain^®^ or Evone^®^). Ventrain^®^ is a manual system utilizing high-pressure oxygen with active expiratory assistance based on the Venturi principle [23], whereas Evone^®^ is a dedicated ventilator allowing FCV. Figure 1 provides a schematic illustration of the Tritube^®^ setup, detailing its specific components and the interface with the Evone^®^ ventilator.

Although these technologies offer an alternative to jet or conventional ventilation, current evidence is still limited, and their applicability depends strongly on equipment availability, operator expertise, and patient selection.

The presented airway management devices available for laryngotracheal surgery are summarized in Table 1.

## 5. Ventilation Issues in Laryngotracheal Surgery

In spontaneous breathing and conventional mechanical ventilation, expiration is a totally passive phenomenon, generated solely by natural elastic recoil of the respiratory system. Thus, both in volume-controlled ventilation (VCV) and pressure-controlled ventilation (PCV), the expiratory phase starts with a peak expiratory flow that progressively decays as airway and alveolar pressures equilibrate. The main limitations to expiration are airway resistance and ID of the ETT, since they reduce the expiratory flow, and together they determine the time required to achieve complete lung emptying before the next cycle [24].

Most of the patients undergoing laryngotracheal surgery are chronic heavy smokers, suffering from COPD. This disease is characterized by chronic small-airway inflammation, sometimes associated with lung emphysema, leading to increased airway resistance and lung elastance, loss of elastic recoil and heterogeneous lung compliance. These result in prolonged expiratory time constants and a strong predisposition to dynamic hyperinflation [25,26].

According to Poiseuille’s law, the resistance is directly proportional to the length of the conduit (i.e., the ETT) and inversely proportional to the fourth power of its radius. As a consequence, reducing the diameter of the ETT to improve the vision of the surgical field significantly and inevitably worsens expiratory flow limitation, as observed with MLTs and laser-resistant ETTs. Therefore, to avoid the risk of air trapping and ensure adequate alveolar emptying, the anesthesiologist in charge must carefully adjust ventilatory settings by lengthening the inspiratory-to-expiratory (I:E) ratio to 1:2 or more and avoiding high respiratory rates, since these will multiply the ventilatory dead space and shorten time for expiration [27].

If the expiratory phase is incomplete, even minimal residual gas volume at the end of each cycle will progressively build up, leading to dynamic hyperinflation. This results in rising airway pressures, reduced inspiratory volumes (due to pressure limit of the ventilator), hypoventilation with hypoxia and hypercapnia, increased risk of barotrauma and pneumothorax, and—in severe cases—hemodynamic compromise caused by impaired venous return to the heart [28]. These mechanisms explain why, despite appropriate ventilatory settings, MLTs and small-bore laser-resistant ETTs may fail to guarantee safe ventilation, leading to potential abortion of the surgical procedure.

The use of ultrathin ETTs introduces specific physiological and technical implications for ventilation. Devices with an ID around 2.4 mm such as the Tritube^®^ make passive expiration inefficient, since the pressure gradient generated by elastic recoil alone is insufficient to overcome the markedly elevated resistance to gas flow as predicted by Poiseuille’s law. For this reason, ultrathin ETTs must be paired with ventilation systems capable of providing active expiratory assistance, such as Ventrain^®^ or the dedicated Evone^®^ ventilator.

**Figure 2:**
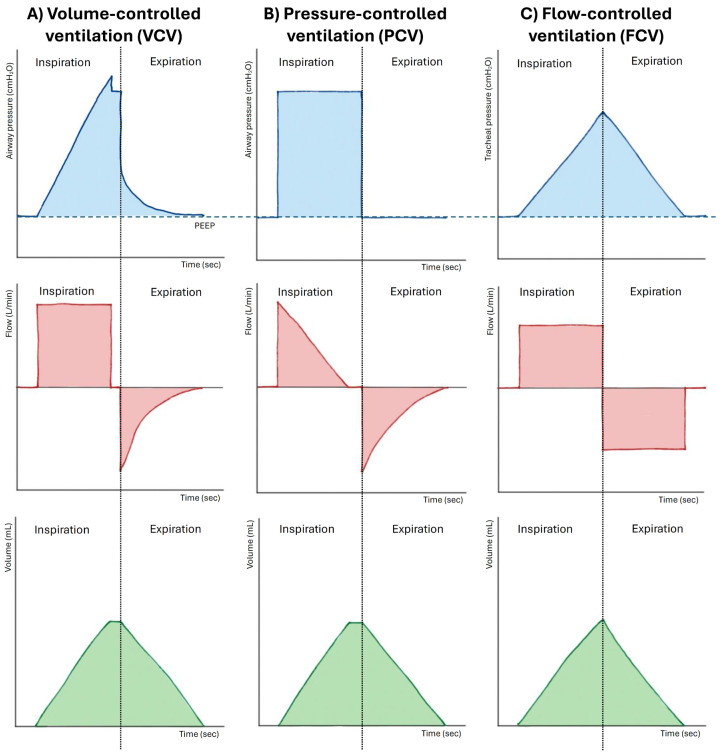
This is a comparative figure detailing the pressure-time, flow-time, and volume-time waveforms and providing a direct visual comparison between Flow-Controlled Ventilation (FCV), Volume-Controlled Ventilation (VCV), and Pressure-Controlled Ventilation (PCV). PEEP: positive end-expiratory pressure.

FCV, delivered by the Evone^®^ platform, differs from conventional modes (VCV and PCV) and HFJV by delivering constant and precisely regulated inspiratory and expiratory flows, resulting in a linear pattern of pressure and volume changes throughout the respiratory cycle, without the pauses and abrupt transitions typical of traditional ventilation. Unlike HFJV, which relies entirely on passive expiration through a narrowed airway and is therefore prone to air trapping, in FCV, expiration is not passive but actively supported through a negative-pressure mechanism based on the Venturi principle, assisting alveolar emptying even when airway resistance is high. This mechanism helps maintain airway patency during expiration, delays airway closure, and promotes a more homogeneous distribution of ventilation, improving gas exchange and reducing atelectasis formation. Figure 2 illustrates the comparative pressure-time, flow-time, and volume-time waveforms for VCV, PCV, and FCV.

FCV requires a sealed airway, standard ASA monitoring with continuous capnography, continuous tracheal pressure monitoring, objective neuromuscular blockade monitoring, and total intravenous anesthesia (TIVA). Given the tiny ID of the ETT, it is relatively contraindicated in case of thick respiratory secretions or bleeding, which can obstruct the lumen. In case of cuff rupture or during emergence from anesthesia, HFJV can be applied through the Tritube^®^ by the ventilator.

In FCV, the anesthesiologist sets FiO_2_, inspiratory flow, I:E ratio, end-expiratory pressure (EEP), and peak inspiratory pressure (PIP). Tidal volume and respiratory rate emerge from the interaction between the set flow rate and the patient’s respiratory mechanics, particularly lung compliance and driving pressure between the set EEP and PIP. Minute ventilation depends on the chosen flow rate and I:E ratio. Moreover, by accurately setting and controlling PIP and EEP, FCV enables targeted management of driving pressure, potentially reducing the mechanical power transmitted to the lungs [29]. These characteristics may be advantageous in obstructed or stiff lungs, where heterogeneous compliance often causes shear forces, pendelluft phenomena, and inefficient ventilation with conventional modes [30].

While the physiological rationale for FCV is compelling, clinical evidence remains limited. In 2020, in two different small, randomized crossover trials, Weber et al. demonstrated that compared with VCV, FCV improved regional ventilation of the lungs and also improved oxygenation and CO_2_ removal at comparable EEP, tidal volume, plateau pressure, and ventilatory frequency [31,32]. A small German preclinical study on 19 pigs showed that FCV attenuated lung injury, enhanced lung aeration in the dependent lung region, and consequently improved gas exchange [33]. In a clinical case report, Barnes et al. calculated the energy dissipation during FCV on a healthy volunteer, showing a lower energy dissipation within the respiratory system than in spontaneous breathing, suggesting that this may have implications in lung protective ventilation strategies [34,35].

Therefore, existing evidence remains limited and heterogeneous, with most studies involving small cohorts, physiological studies, or animal models, highlighting the need for larger prospective trials to clarify the full clinical impact and safety profile of FCV.

Clinical data regarding the use of ultrathin ETTs and FCV in laryngeal surgery are limited to case reports and small case series. With their obvious limits, they indicate feasibility, with optimal oxygenation and CO_2_ trends and generally low driving pressures. Reported complications include tube displacement, kinking, obstruction from secretions, and occasional ventilator malfunction requiring system reset [11,12,13]. These limitations underscore the need for careful patient selection and operator familiarity with the equipment.

Beyond laryngotracheal procedures, FCV has been explored in other surgical settings, including thoracic, robotic, and cardiac surgery. In a small, single-center, randomized clinical trial, Abram et al. demonstrated higher PaO_2_/FiO_2_ ratios, lower minute ventilation, and lower mechanical power with better gas exchange using FCV compared with PCV in one-lung ventilation, suggesting potential benefits beyond airways surgery [36].

All ventilation techniques that require deep neuromuscular blockade (e.g., for placement of ultrathin ETTs and to facilitate a completely still surgical field) also necessitate adequate depth of anesthesia and strict control of muscle relaxation. In accordance with current safety guidelines, the use of objective neuromuscular monitoring—e.g., using a Train-of-Four (TOF) device—is mandatory [37]. This practice ensures profound blockade is maintained when required and, more critically, that complete recovery of neuromuscular function (TOF ratio > 0.9) is confirmed before any attempt at extubation, thereby mitigating the risk of residual neuromuscular blockade (RNMB), which is particularly critical in patients with airways that have been surgically manipulated or compromised [38].

## 6. Extubation and Postoperative Airway Management

Extubation after laryngotracheal surgery requires careful planning, as postoperative airway edema, residual bleeding, and altered laryngeal dynamics may significantly compromise patency. A staged approach is often recommended, including a meticulous assessment of airway patency. This assessment should feature the Air Leak Test (ALT)—performed by deflating the cuff and measuring the difference between inspiratory and expiratory tidal volumes—as a quantitative screen for significant laryngeal edema [39]. ALT should be coupled with endoscopic inspection of the glottic and subglottic region and ensure complete reversal of neuromuscular blockade with TOF monitoring [40]. In patients with pre-existing stenosis, recent dilation, or extensive mucosal manipulation, delayed extubation or short-term postoperative airway support (CPAP or HFNC) may reduce the risk of early obstruction.

Postoperative monitoring must be tailored to the underlying pathology and the extent of the procedure. Patients with significant airway narrowing, COPD, or those who require prolonged jet ventilation or FCV may experience impaired clearance of secretions or dynamic collapse, necessitating observation in a high-dependency unit. Effective coordination between anesthesiologists and ENT surgeons is essential to anticipate complications and promptly secure the airway if deterioration occurs.

## 7. Conclusions

Airway management and ventilation during laryngotracheal surgery remain among the most complex and delicate tasks in anesthesiology. The need to ensure optimal surgical exposure while simultaneously maintaining adequate oxygenation and ventilation represents a constant challenge for anesthesiologists. Traditional strategies, such as intermittent apneic oxygenation and JV, still play a role in many clinical contexts but are often associated with limitations, including inadequate ventilation, barotrauma, or interference with surgical access.

More recent techniques, including the use of ultrathin ETTs and flow-based ventilation strategies, have introduced new opportunities for balancing surgical and anesthetic requirements, offering improved gas exchange and enhanced safety. Nevertheless, these techniques require specific expertise, the availability of equipment, and close interdisciplinary collaboration. Current evidence, while encouraging in selected settings, is still limited and heterogeneous, underscoring the need for cautious interpretation and further research.

Given the variability in airway anatomy, pathology, and surgical requirements, the most effective strategy is rarely universal; instead, a tailored approach remains essential. Optimal management depends on a clear understanding of the strengths and limitations of each technique, proactive planning for contingencies, and close coordination between anesthesiologists and surgeons. Table 1 summarizes the different advantages and drawbacks, as well as the indications and safety concerns, of different airway management devices and can be used to guide what device to use according to different clinical scenarios.

Continued technological development, alongside well-designed prospective studies, will be essential to better define the role of emerging techniques and to support evidence-based recommendations. Ultimately, maintaining flexibility and adopting a patient-centered approach remain key determinants of safety and success in this complex field.

## Figures and Tables

**Table 1:** Airway management devices used for laryngotracheal surgery.

Technique	Main Advantages	Main Limitations	Typical Indications	Key Safety Concerns
**High-Frequency Jet Ventilation (HFJV)**	Minimal or no obstruction of the surgical field (“tubeless” technique); Effective oxygenation with high-frequency pulses; Can be used to ventilate through cricothyroidotomy cannula or rigid bronchoscopy	Poor control of minute ventilation; Needs an open and unobstructed airway: not suitable for near-occlusive stenosis; Risk of air trapping	Posterior commissure surgery; Moderate stenosis; Laser procedures requiring good exposure (not achievable with ETT)	Barotrauma, pneumothorax; Dynamic hyperinflation; Hemodynamic decompensation; Respiratory acidosis in low-compliance lungs and long procedures
**Apneic Oxygenation and Intermittent Apnea**	Fully tubeless field; Simple and widely available; Optimal and unobstructed vision for surgeon	Rapid hypoxia and accumulation of CO_2_; Poor option in low reserve patients	Very short procedures; Laser microdebridement; Quick balloon dilations	Severe desaturation if preoxygenation is inadequate; Risk of CO_2_ accumulation in prolonged apnea; Risky in obesity/OSAS
**Microlaryngeal Tubes (MLTs) and Laser-Resistant ETTs**	Easy to use, standard technique; Widely available; Allow airway protection and full ventilatory control; Good in high-risk respiratory patients	Narrows surgical field, can obstruct laser access; Impediment to posterior commissure; Higher expiratory flow resistance, with risk of hyperinflation in COPD; Cuff vulnerable to laser	Routine laryngeal surgery; Long procedures in patients with low functional reserve who would not tolerate HFJV or intermittent apnea	Long expiratory time constants with need for longer expiratory times; Risk of dynamic hyperinflation; High ventilatory pressures with risk of barotrauma; Fire risk if laser is used (need to use FiO_2_ < 30%)
**Tritube**^®^ **+ Flow-Controlled Ventilation (FCV)**	Optimal surgical exposure; Active expiration improves lung recruitment and CO_2_ removal; Predictable, stable ventilation; Continuous tracheal pressure monitoring	Limited availability and high costs; Requires Evone^®^ ventilator and dedicated consumables; Not ideal with thick secretions; Requires appropriate training	Laryngeal and posterior commissure lesions; Subglottic and tracheal stenosis; Long procedures needing tight ventilatory control and adequate ventilation	Tube obstruction (e.g., by secretions or kinking); Loss of seal (in case of cuff rupture); Ventilator malfunction; Not laser-safe tube (the cuff needs to be protected)
**Cuirass/Negative-Pressure Ventilation**	Completely tubeless; Useful in fragile or injured airways	Limited availability; Ineffective with obesity or stiff chest wall; Bulky; may hinder access	Fragile airway mucosa with risk of bleeding; Rare selected glottic/subglottic stenoses, where an ETT would impede surgical access	Pressure ulcers caused by the rigid cuirass shell; Incomplete ventilation if inadequate seal or reduced compliance; Unprotected airway with risk of aspiration; Hemodynamic decompensation and right heart strain; CO_2_ and minute volume are difficult to monitor with an open airway

## Data Availability

No new data were created or analyzed in this study. Data sharing is not applicable to this article.
